# Editorial: Insights in renal and epithelial physiology: 2023

**DOI:** 10.3389/fphys.2024.1523820

**Published:** 2024-12-10

**Authors:** Carolyn M. Ecelbarger, Xiaoyan Zhang, Youfei Guan

**Affiliations:** ^1^ Department of Medicine, Division of Endocrinology and Metabolism, Washington, DC, United States; ^2^ Health Science Center, East China Normal University, Shanghai, China; ^3^ Advanced Institute for Medical Sciences, Dalian Medical University, Dalian, Liaoning, China

**Keywords:** lithium, allograft acceptance, biomarkers, nutriceutical agents, calcium signal, acute kidney damage

We were overall delighted with the high-interest generated in this second iteration of Insights in Renal Epithelial Physiology encompassing 2023–2024. We were pleased to accept 9 reviews, primary reports, and a meta-analysis on wide-ranging Research Topic from renal cell signaling mechanisms and approaches to study them, novel therapeutics and nutrition-based strategies to combat renal injury, and updates on sensitive and specific early biomarkers for renal disease. We highlight these reports below.

Lithium, used primarily in psychiatric therapy, has been associated with renal injury (Kishore and Ecelbarger, 2013). Baranovskaya et al. assessed the extent to which inflammation contributed to overall damage using a mouse model of lithium carbonate treatment. They conducted measures of renal injury including, terminal deoxynucleotidyl transferase dUTP nick end labeling (TUNEL) assay, kidney injury marker (KIM1) and neutrophil gelatinase lipocalin (NGAL). In addition, they assessed activation of the pro-inflammatory nucleotide-binding domain, leucine-rich–containing family, pyrin domain–containing-3 (NLRP3) inflammasome cascade. They determined that lithium induced activation of apoptosis, but not general inflammation, in fact inflammation was somewhat reduced (lower M1/M2 polarization ratio, caspase-1, NLRP3, and interleukin 1β levels) in the lithium-treated mice. Overall, they concluded that while lithium did insult the kidney primarily via apoptosis; the anti-inflammatory effect may be beneficial.

Guo et al. provide a mini-review on the role of bile acid receptors in water homeostasis highlighting what is known regarding the farnesoid X receptor (FXR) and the Takeda G protein-coupled receptor 5 (TGR5). While these receptors have important role in renal metabolism, less is known regarding their impact on urine concentration. FXR is a nuclear receptor primarily involved in regulating transcription, whereas, TGR5 couples to Gs-alpha (Gαs) which activates the cAMP-protein kinase A signaling (PKA) pathway. Zhang et al. (Zhang et al., 2014) showed that urine became more concentrated in mice treated with bile acids, and FXR knockout mice had impaired urine concentrating ability. In addition, activation of FXR seems to reduce renal cell apoptosis under hypertonic stress (Xu et al., 2018), and enhance activity of the sodium coupled Na-K-2Cl cotransporter (NKCC2) in the thick ascending limb. With regard to TGR5, Han et al. (Han et al., 2021) showed increased expression of aquaporin 2 in an ischemia/reperfusion model of impaired urine concentration most likely via activation of the hypoxia-inducible factor (HIF) pathway. These findings suggest some potential therapeutic benefits of bile acids in urine concentration.

Sickle cell disease (SCD) is associated with sickle cell nephropathy (SCN) likely due to poor perfusion of capillaries with the malformation of red blood cells (Ataga et al., 2022). Early detection of nascent SCN is critical in staving off pathology. Packialakshmi et al. conducted proteomics followed by Western blotting on urine exosomes to look for early biomarkers of SCN in humanized sickle-cell disease (SCD) mice pre- and post-development of albuminuria. Potential early biomarkers they detected that correlated with albuminuria in the mice were heparanase, cathepsin C, α2-macroglobulin, and sarcoplasmic endoplasmic Ca^2+^ATPase-3 (SERCA3). Female mice demonstrated a stronger correlation of these proteins with albuminuria. These studies provide candidates to assess in human subjects.

It is important to predict, as early as possible, whether a renal allograft will be successful. Kidney biopsies have complications and are often not reliable. Yang et al. conduct a meta-analysis to assess the diagnostic performance of graft-derived cell-free DNA (GcfDNA) in determining kidney allograft rejection rates. Eleven studies from four continents comprising 1,148 patients were statistically evaluated. GcfDNA was found to be useful particularly as a biomarker for discriminating between rejection and antibody-mediated rejection (ABMR) in transplant recipients.

Tuberous sclerosis complex (TSC) genes 1 and 2 (coding for harmatin and tuberin, respectively) are important cell growth regulators. Mutations in their function (also known as TSC), leads to cystic growth in kidney and other tissues. Soleimani reviews manifestation of this disorder in kidney and delineates how it differs from other major cystic disorders, e.g., polycystic kidney disease. Normally functioning TSC brakes over-activity of the mechanistic target of rapamycin (mTOR), upstream of growth and cell proliferation. In kidney, there appears to be involvement particularly in alpha intercalated cells via FOXI1 and upregulation of H + -ATPase signaling. Thus, there is potential for refined therapeutic targeting in TSC-associated renal cysts.

Zou provides a review on advances in the use of microRNAs to predict cardiovascular complications in chronic kidney disease (CKD). Reviewing animal and human studies, they highlighted a number of miRs that have been reported to be associated changes in vascular calcification or ventricular hypertrophy, e.g., miR-29b, miR-378-3p, and miR-30. Tables were provided showing candidate miRs and main findings. Development of a circulating prognostic panel of miRs in this patient population would move the field forward and help improve patient outcomes.

Hyperuricemia is an increasing disorder associated with metabolic syndrome, likely due to high purine and fructose metabolism (Yanai et al., 2021). Umer et al. used a potassium oxonate/bromate model of hyperuricemia in rats to study the potential therapeutic role of onion bioactive compounds, e.g., quercetin. Oral onion powder significantly reduced blood uric acid levels, in dose-wise fashion, and improved liver and kidney function and lipid profile. There were no effects on weight gain. The absolute component of the onion powder that improved metabolic profile in the rats was not determined, but these findings provide rationale for further study of underlying mechanisms.

Simpler, and more easily manipulated, model systems improve our ability to understand complex signaling in the kidney. Calcium mobilization in inner medullary collecting duct cells has been shown to be downstream of G-protein coupled signaling via exchange proteins directly activated by cyclic AMP (Epac). Using fluorescence and site-specific Ca^2+^ sensitive biosensors, Yip et al. evaluate whether murine principal kidney collecting duct cells (mpkCCD) are a reliable model for intact collecting duct with regard to Ca^2+^ mobilization characteristics. It proved reliable and they elucidated the nature of ryanodine-dependent Ca^2+^ signaling and endoplasmic reticulum (ER) mitochondrial Ca^2+^ coupling in this system.

Acute hemorrhagic events followed by necessary staunch of blood loss with a tourniquet can lead to acute kidney injury. To better treat and avoid this event, appropriate animal models are needed. Packialakshmi et al. studied the impact of hemorrhage coupled to tourniquet (lower-limb) application using a mouse model. They found hemorrhage alone did not lead to AKI (15% blood loss), but the coupling with ischemia (tourniquet) significantly exacerbated renal, as well as, lung injury associated with the ischemia. The new model system can be employed to evaluate mechanisms and therapeutic strategies.
